# Localized direction selective responses in the dendrites of visual interneurons of the fly

**DOI:** 10.1186/1741-7007-8-36

**Published:** 2010-04-12

**Authors:** Christian Spalthoff, Martin Egelhaaf, Philip Tinnefeld, Rafael Kurtz

**Affiliations:** 1 Department of Neurobiology, Bielefeld University, Postbox 100131, 33501 Bielefeld, Germany; 2 Angewandte Physik - Biophysik, Ludwig-Maximilians-Universität, Amalienstrasse 54, 80799 München, Germany

## Abstract

**Background:**

The various tasks of visual systems, including course control, collision avoidance and the detection of small objects, require at the neuronal level the dendritic integration and subsequent processing of many spatially distributed visual motion inputs. While much is known about the pooled output in these systems, as in the medial superior temporal cortex of monkeys or in the lobula plate of the insect visual system, the motion tuning of the elements that provide the input has yet received little attention. In order to visualize the motion tuning of these inputs we examined the dendritic activation patterns of neurons that are selective for the characteristic patterns of wide-field motion, the lobula-plate tangential cells (LPTCs) of the blowfly. These neurons are known to sample direction-selective motion information from large parts of the visual field and combine these signals into axonal and dendro-dendritic outputs.

**Results:**

Fluorescence imaging of intracellular calcium concentration allowed us to take a direct look at the local dendritic activity and the resulting local preferred directions in LPTC dendrites during activation by wide-field motion in different directions. These 'calcium response fields' resembled a retinotopic dendritic map of local preferred directions in the receptive field, the layout of which is a distinguishing feature of different LPTCs.

**Conclusions:**

Our study reveals how neurons acquire selectivity for distinct visual motion patterns by dendritic integration of the local inputs with different preferred directions. With their spatial layout of directional responses, the dendrites of the LPTCs we investigated thus served as matched filters for wide-field motion patterns.

## Background

Fast flying insects such as flies need to integrate motion signals from their compound eyes for various tasks which include course control [1], collision avoidance and the detection of small objects. These tasks require that signals from individual ommatidia be combined into local motion signals, a process which is thought to take place in the medulla [2]. Local motion signals are then pooled and integrated on the dendrite of cells, which receive input from thousands of ommatidia. In the blowfly, this integration of retinotopic information occurs in the lobula plate, with a class of about 60 individually identifiable neurons showing direction selective responses to visual stimuli, the lobula-plate tangential cells (LPTCs). Among the best characterized of these cells are the neurons of the so called 'horizontal system' (HS), named after their strong response to progressive (front-to-back) motion, and those of the 'vertical system' (VS), which respond mainly to downward motion. However, similar to many large-field neurons in other species [3,4], the motion direction that evokes maximal responses is not uniformly the same but varies in the different parts of the visual field. This led to the conclusion that several LPTCs are tuned to a particular type of optic flow: visual motion patterns like those encountered during specific flight manoeuvres [5-7]. It is known that many LPTCs receive input from local motion-detecting elements on their dendritic trees in a fundamentally retinotopic manner [8,9]. Little is known, however, about the fine-scale structure of these input signals. In particular, it remains unclear how the variations in local preferred directions across the large receptive fields are structurally represented.

Due to their small size, the local input elements of LPTCs do not lend themselves well to electrophysiological recording and there is little direct evidence for their responses to motion [10]. In order to circumvent this limitation, we measured localized calcium concentration changes at the dendrites of LPTCs. Ca^2+ ^entry into the dendrite results partially from the activity of voltage-gated calcium channels [11,12] and concentration changes of Ca^2+ ^have been shown to remain local [9,13,14]. Consequently, fluorescent calcium-sensitive dyes can be used as a general indicator of potential changes in the local dendritic membrane while simultaneously allowing the visualization of the dendritic fine structure.

In the present study we monitored the local direction tuning of dendritic calcium signals and compared it to that obtained by axonal voltage recordings in different types of LPTCs. The VS consists of 10 neurons per brain hemisphere, which respond predominantly to vertical motion within large, dorso-ventrally elongated sections of the visual field. It is a remarkable feature of VS neurons that their axonal voltage responses are made up of two components: Either graded changes in membrane potential, which consist of depolarization during motion in one direction (the 'preferred direction') and hyperpolarization during motion in the opposite direction (the 'antipreferred direction') or modulations in the rate of spikes. Rather than being all-or-none by nature these spikes may vary in their amplitude [15]. In contrast to the VS neurons, which show a mixture of spiking and graded responses, the second type of LPTC analysed in the present study - the 'ventral centrifugal horizontal'cell (vCH) - responds with purely graded voltage responses to visual motion. It receives input from the ipsilateral field of view via electrical synapses formed with the dendrites of HS cells [16] and is thus most sensitive to horizontal motion. We present data for a third type of LPTC which has so far not been physiologically characterized in a systematic way. Based on similarity with anatomical data presented in [17] we classified it as 'Amacrine cell' (called hereafter 'Amx').

In our study we aimed to investigate how the intricate layouts of the receptive fields of LPTCs, which distinguish themselves by highly specific patterns of local preferred directions, are formed by dendritic input integration. We find that dendritic activation patterns, as monitored by calcium imaging, reflect a retinotopic projection of local inputs from the visual field onto the dendrite of individual neurons. This subcellular representation of inputs is similar to, for example, the retinotopy that is present on a multi-neuron level in the mammalian primary visual cortex. Thus, local variations of preferred directions, which are present across the receptive fields of LPTCs [6,7], are also expressed across their dendrites. However, dendritic calcium activation patterns are further shaped by indirect non-retinotopic inputs, which modify direction specificity in subregions of the dendrite.

## Results and discussion

### Axonal direction tuning and extent of dendritic activation

As a mechanism underlying the specificity of individual LPTCs for particular patterns of optic flow, it has been presumed that LPTCs sample local inputs with different preferred direction across their dendrites [6,7]. This conclusion is mainly based on axonal recordings which provide a limited insight into the actual process of dendritic information processing. In order to fill this gap, we monitored local activity levels and determined the local preferred directions in the dendritic trees of several types of LPTCs.

**Figure 1:**
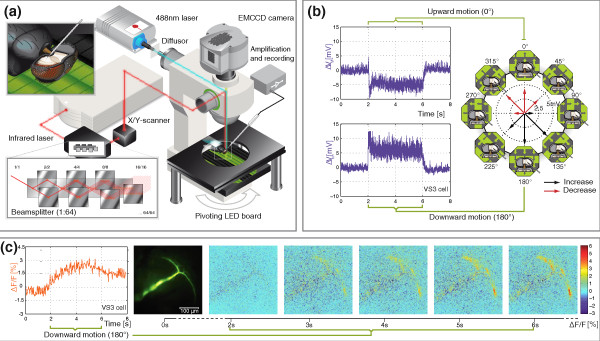
**Acquisition of voltage and calcium responses to visual motion**. (a) Setup for simultaneous electrophysiology and calcium imaging by multifocal two-photon and conventional wide-field microscopy. Insets show the recording site (top left) and the multifocal beamsplitter for two-photon microscopy (bottom left). The motion stimulus consists of a drifting square wave grating generated by a light emitting diode board. (b) Response of a vertical system (VS)2/3 neuron to a grating moving in preferred (bottom left) and antipreferred (top left) direction and directional tuning (right). Arrow directions indicate the direction of motion; arrow lengths represent response amplitudes averaged over an interval of 4 s starting at motion onset minus the mean response during 2 s before motion onset. Black arrows signal increases (depolarization); red arrows signal decreases (hyperpolarization). (c) Calcium response to downward motion of the same VS cell stained with Oregon Green BAPTA-1. Time course of the calcium signal integrated over the whole dendrite (left) and series of colour-coded Δ*F*/*F*_0 _images showing local differences in fluorescence intensity for various time points (right). Resting fluorescence *F*_0 _was determined by averaging the last three frames before start of pattern motion. Images were taken at 10 Hz and 512 × 512 pixel resolution.

As a reference for our measurements of dendritic motion sensitivities, we first investigated the global spatially integrated direction selectivity in the output region of various LPTCs to our set of visual stimuli, which consisted of grating patterns moving in different directions in the frontal visual field. Figure 1 shows the setup, recording site and stimulation pattern (a) and gives an example of an intracellular recording from a VS2 or VS3 neuron during the presentation of a square wave grating (octagon with an extent of -40° to +40° in elevation and in azimuth) drifting upward (0°, Figure 1(b), top left) or downward (180°, bottom left). Since VS2 and VS3 are difficult to differentiate on an anatomical basis and exhibit very similar response properties, we will refer to them together as VS2/3 from now on. The VS2/3 cell responds with a combination of spikes and graded membrane depolarizations to downward motion and with hyperpolarization to upward motion (Figure 1(b)). The global direction preference of the neuron was determined by measuring the responses to eight motion directions. The response amplitudes, plotted as vectors in Figure 1(b), show a symmetrical, nearly sinusoidal, direction tuning with a resulting preferred direction at ~180° for the VS2/3 cell, consistent with what is known from earlier studies [6].

In order to visualize spatially resolved dendritic activity, the cells were filled with the fluorescent calcium-sensitive dye Oregon Green BAPTA 1 (see Methods). The calcium responses show that distinct areas of the dendrite were activated by a downward motion (Figure 1(c)). While the axon displays only weak calcium signals, the dorsal and ventral dendritic tips show strong increases in calcium. Our stimulus covered only part of the entire field of view of the fly's compound eye (-40° to 40° out of +90° to -75° in elevation and -5° to 40° out of 5° to >180° in azimuth for a single eye). Nonetheless, the calcium responses were expressed over almost the entire vertical extent of the dendrite, the tips of which nearly reach the dorsal and vertical rim of the lobula plate. Given the retinotopic layout of the lobula plate, this result implies that a disproportionately large amount of space is devoted to the processing of visual information from the frontal visual field, similar to the fovealization in the visual system of many vertebrates.

### Local dendritic directional preferences

**Figure 2:**
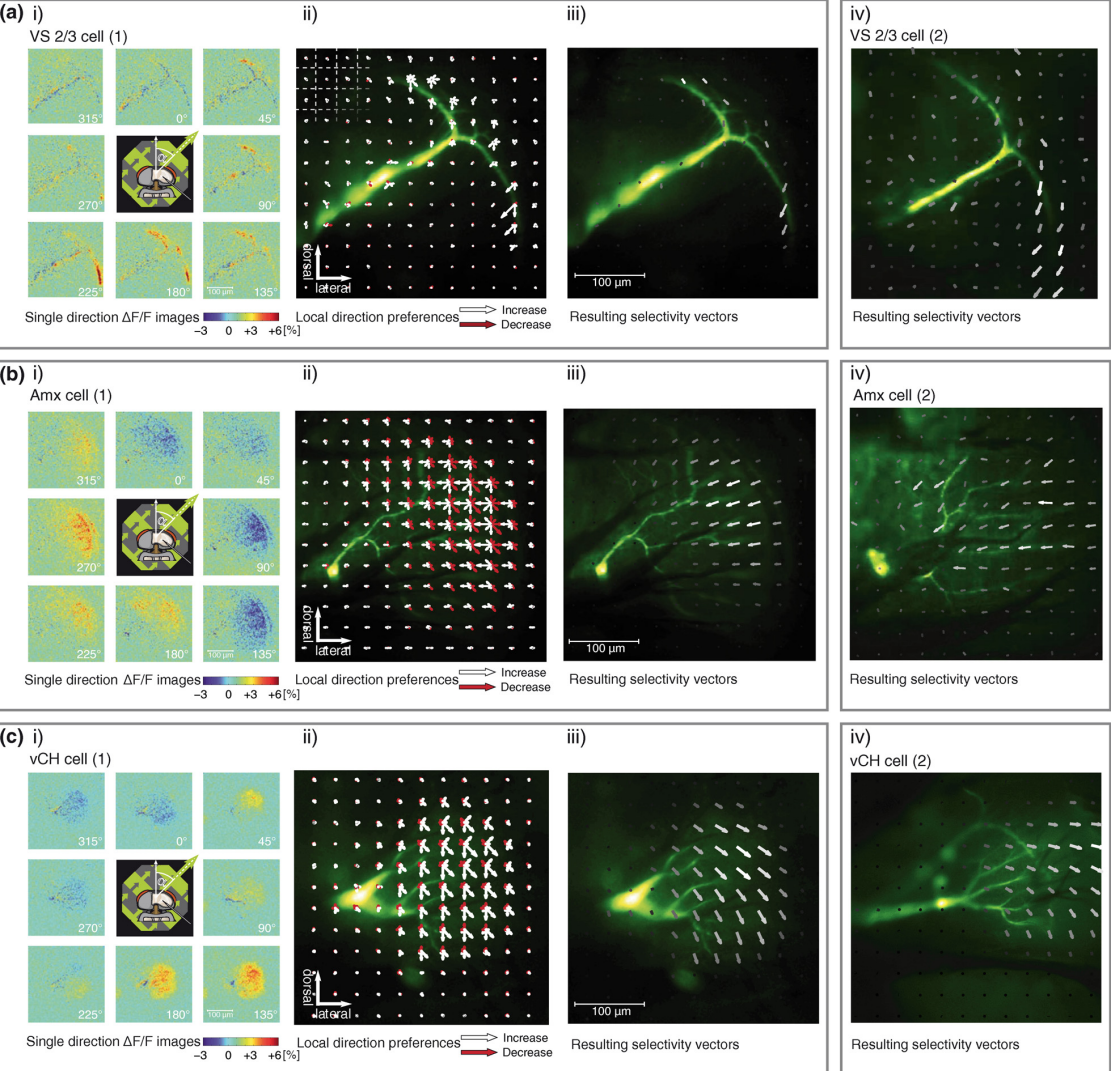
**Local dendritic directional preferences of various lobula plate tangential cells (LPTCs)**. Calcium signals at the dendrites of three different LPTC classes: vertical system (VS)3 cells (a), neurons called Amacrine cell (b) and ventral centrifugal horizontal cells (c). (i) Local differences in fluorescence intensity recorded in a single cell during pattern motion in eight directions (α) in comparison to an average of the last three frames before onset of motion. Signals were averaged over the last 500 ms of stimulus motion. (ii) Integrated local calcium responses to pattern motion in eight directions - the same cell as in (i). The grid of region of interest (ROIs) is indicated in the upper left corner. White arrows show increases in calcium in the underlying ROI in response to motion in the direction of the arrow; red arrows show decreases. Arrow length (normalized to the arrow with maximum amplitude in the image) represents Δ*F*/*F *intensity. (iii): Resulting response vectors from vector summation of the individual response vectors to all stimulus directions (normalized as before) - the same cell as in (i). Arrow brightness represents overall response amplitude. (iv) Resulting response vectors of additional cells of each of the cell types recorded in different flies, calculated as in (iii). In (c), right column, the profile faintly visible in the dorsal area is a VS cell which was accidentally stained during tissue penetration. The cell did not noticeably contribute to the calcium signals. Images represent single recording traces and were taken at 10 Hz and 512 × 512 px resolution.

In order to investigate how variations in motion preference in the visual field are represented in the local activation patterns of the dendrite, we recorded calcium image series from different types of LPTCs while we presented motion in eight directions [Figure 2, first column (i)]. In order to visualize local direction preferences for motion in the different regions of the dendrite, the complete image series were divided into a grid of 16 × 16 square regions of interest (ROIs) comprising 32 × 32 pixels each. The relative fluorescence change in each ROI was then pooled and summed to a scalar value. This set of response amplitudes for each ROI is displayed as a set of eight arrows placed at the centre of each ROI, with length corresponding to amplitude (negative values are plotted in red) and direction to stimulus direction, and the plots are overlaid on the raw fluorescence image of the cell [Figure 2, second column (ii)]. In order to calculate selectivity and preferred direction for each ROI, these eight vectors were added to a single resulting vector and plotted as arrows, with the arrow direction and length corresponding to the directional preference (see Methods) and the arrow brightness to the overall signal intensity [Figure 2, third and fourth columns (iii and iv)]. All cells examined show calcium signals over almost the whole extent of the dendritic tree but there are distinct variations in their local dendritic directional preferences.

In VS2/3 cells (examples shown in Figures 1(c) and 2(a)), the calcium signals mainly consist of increases, with no signal decreases in the dendritic branches. This effect was attributed to the fact that, during antipreferred direction stimulation, the calcium signal could consist not only of a concentration decrease due to closure of voltage-gated calcium channels but - at least close to synaptic input sites - also of an influx of calcium. The latter might result from a calcium influx through ACh receptors, which are thought to be Ca^2+ ^permeable and slightly activated during motion in antipreferred direction [18]. Local preferred directions across the dendrite of VS2/3 reveal a curving pattern, with horizontal and downward/front-to-back selectivities predominant in the dorsal dendritic branch, purely downward selectivities near the major dendritic branching point and downward/back-to-front selectivities in the ventral branch. This pattern matches a retinotopic representation of the response field of VS2/3 measured by axonal voltage recordings during presentation of spatially confined stimuli [6]. In Figure 2(a) another example of a VS2/3 recording is shown. The overall signal strength was weaker in this cell, which leads to increased background noise and weak signals in the dorsal dendritic branch. However, the ventral dendrite shows the same curving pattern of the direction selectivities as the first example. A similar response profile could be recorded in a third VS 2/3 cell (data not shown).

We also recorded calcium and voltage signals from two cells which we called Amx cells based on their anatomy. Similar to the Am1 cell [17], these cells have no prominent axon and their dendrites cover most of the lobula plate, showing a distinct double band of fine arborizations at and beyond the rim of the lobula plate [Figure 2(b), (i-iv)]. In voltage recordings, these cells displayed mainly graded responses, with a preference for back-to-front motion (data not shown). The direction selectivities of spatially resolved calcium signals of Amx cells differ considerably across the dendrite, with dorsal and ventral selectivity patterns smoothly changing from a 225° preference in the dorsal dendrite to a 270° preference in the medial and a 315° preference in the ventral dendrite. Motion in the local antipreferred direction led to strong decreases in the local calcium signals, but the relation of the amplitude of increases and decreases was inhomogeneous in different parts of the dendritic tree: calcium concentration decreases were particularly pronounced in medial parts of the dendrite. While the extent and response pattern of the field of view of this cell has not yet been characterized, the dendritic response field would suggest that the cell responds with strong hyperpolarization to an expanding flow field with a centre of expansion directly in front of the animal, a response characteristic which has not yet been found in other LPTCs.

Figure 2(c) shows two examples of a vCH, a cell that receives input from the ipsilateral field of view via electrical synapses formed with the dendrites of HS cells [16]. Unlike the VS neuron shown before, but similar to the Amx cell, vCH shows not only increases in calcium concentration during the preferred direction motion, but also decreases in response to motion in its antipreferred direction. This difference between the cell types has already been demonstrated by Dürr *et al*. [19] and might be attributed to the fact that vCH receives ipsilateral input via electrical synapses with HS neurons, so that influx via transmitter-driven channels (see above) has no effect on calcium concentration. HS neurons are primarily activated by a front-to-back motion in the ipsilateral field of view and by a back-to-front motion in the contralateral field of view [20,21]. A corresponding horizontal motion sensitivity of the calcium response field of vCH would be expected if its dendritic responses were dominated by input from HS neurons. However, it is known that vCH also receives input from additional contralateral elements which may modulate the directional selectivity.

**Figure 3:**
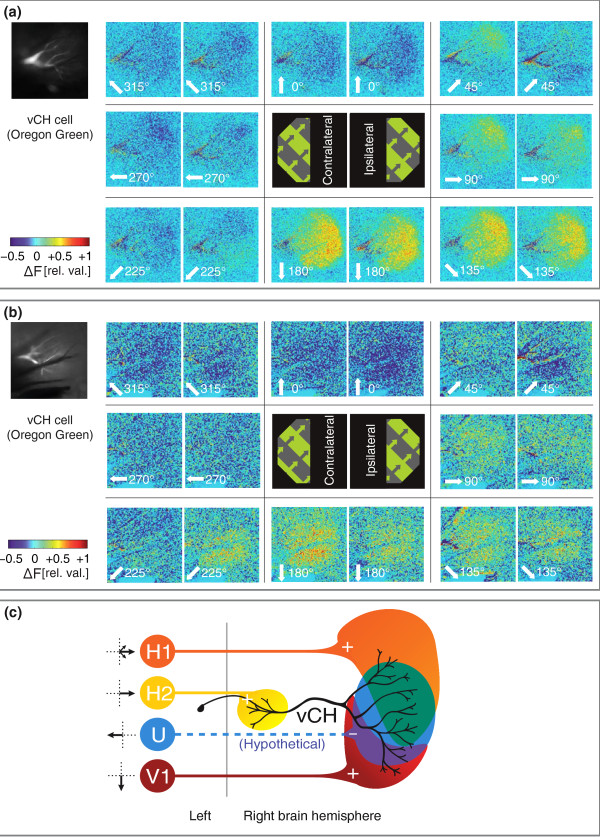
**Influences of ipsi- and contralateral inputs on ventral centrifugal horizontal (vCH) calcium signals**. (a and b) Image pairs showing local differences in fluorescence intensity in a single vCH cell each during pattern motion in eight directions (white numbers), with only the contralateral (left panels) or ipsilateral (right panels) half of the stimulus pattern visible. Signals were averaged over the last 500 ms of stimulus motion. Images represent single recording traces and were taken at 10 Hz and 512 × 512 pixel resolution. (c) Wiring diagram of connections between contralateral lobula plate tangential cells and vCH. Black arrows next to the cells indicate the PD of visual motion in the contralateral receptive field of the cells. +: Excitatory input, -: inhibitory input. Wiring of H1, H2 and V1 after [23].

The dendritic calcium responses of vCH [Figure 2(c), (iii)] show a shift of the selectivity vectors from a front-to-back motion in the dorsal dendrite to a downward motion in the ventral dendrite, with similar results observed in two other vCH cells [one shown in Figure 2(c), (iv)]. This sensitivity to vertical motion was also found in electrical responses by Krapp *et al. *[22]. A plausible reason for this response is the input from the contralateral V1 cell, which shows a strong response to frontal downward motion stimuli and terminates in the ventral half of the lobula plate [22] (see Figure 3(c)). This cell has been shown to be coupled with vCH [23] and may contribute to the strong vertical sensitivity in the ventral part of the dendrite of vCH. This would add a non-retinotopic influence to the motion signals on this part of the dendrite of vCH.

### Influences of ipsi- and contralateral inputs on vCH calcium signals

In order to test how strongly contralateral and ipsilateral inputs are represented in the dendritic calcium signal of the vCH cell, we covered one half of the stimulus area at a time, blocking most of the field of view of either the left or the right eye apart from the narrow region of binocular overlap, which reaches 5-10° into the respective contralateral visual field in female *Calliphora *[24]. Figure 3(a) shows the responses of two vCH cells to motion presented in either the ipsi- or contralateral visual field. The calcium signals show that responses to contralateral stimulation were about equally strong as to ipsilateral stimulation. This may partly be due to the fact that the receptive fields of most inputs to vCH cover the region of binocular overlap. However, as a consequence of retinotopy in its input from the ipsilateral eye, one would expect the responses to contralateral motion to be confined to the lateral part of the dendrite, which receives input from the frontal region of binocular overlap. Such a regional confinement is not present in our measurements. Thus, it is plausible that the strong responses to contralateral stimulation are to some extent caused by contralateral inputs converging on the dendritic tree.

Apart from the V1 mentioned above, sensitivity to contralateral motion might be mediated by the contralateral H1-neuron, which is known to be synaptically coupled with vCH [23]. This cell covers a large part of the dorsal dendrite of vCH with its terminal arborization (see scheme in Figure 3(c)), where responses are markedly stronger during contralateral than during ipsilateral stimulation. Another candidate mediating the decreases in calcium signal during contralateral stimulation, such as those observed in response to 270° and 315° motion, could be the so far unidentified neuron that provides inhibitory input to vCH during contralateral front-to-back motion called 'U' in Krapp *et al*. [22].

Overall, our results imply that, for vCH, contralateral elements not only play a role in the pooled axonal voltage response to wide-field motion as shown in [22], but that the extent and location of the arborizations of these cells can influence the dendritic representation of wide-field motion. For vCH, the layout of dendritic interactions is particularly relevant because the dendrite of vCH is not only an input region but forms a spatially distributed, inhibitory GABAergic synapse [25], which is part of a circuit for figure-ground discrimination [26,27]. How this summation affects the dendritic response of vCH and, thus, the input to FD cells can only be addressed by further investigation with more localized stimuli.

### Fine-scale direction tuning of LPTC dendrites

In order to investigate whether additional fine-scale inhomogeneities in direction tuning originating from individual input elements can be found, we examined several parts of the dendritic tree of a VS1 cell under higher magnification. For example, the pooling of motion signals originating from different pairs of neighbouring ommatidia might take place in locally distinct areas of the dendrite. In addition to the two-dimensional layout of the dendrite, this spatial separation of inputs might also be present in the *z*-direction: In the lobula plate, motion signals from local elements that are sensitive to vertical motion have been argued to terminate in more superficial tissue layers than those sensitive to horizontal motion [28]. In order to reduce cross talk between calcium signals originating from dendritic branches located in different depth layers of the tissue, we confined excitation to a single plane by using two-photon laser scanning microscopy (TPLSM). Our setup made use of a multifocal excitation scheme to enable the acquisition of entire images at a higher temporal rate than is possible with single focus scanning [29].

**Figure 4:**
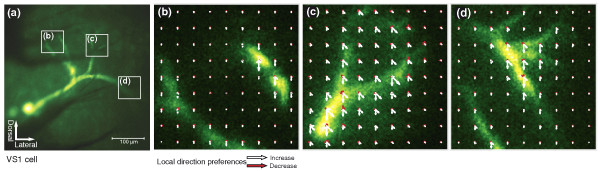
**Fine-scale direction tuning of vertical system (VS)1 cell dendrites**. (a) VS1 cell stained with Oregon Green BAPTA-1 showing the positions of the recording areas B-D. (b) Calcium response field of the dorsomedial dendrite to a square grating moving in eight directions. Presentation as in Figure 3. (c) Response field of the dorsal and (d) of the lateral dendritic branch of the same VS cell. Images b-d were taken at 10 Hz and 256 × 256 pixel resolution with multifocal two-photon laser-scanning microscopy.

Figure 4 shows an image of a VS1 cell (A) and three calcium response fields of small dendritic areas examined with TPLSM (B-D). In all these areas direction tuning appears fairly homogeneous over the whole extent of the dendrites. Slight shifts in the preferred direction are present within the imaged regions, but abrupt changes from one branch location to the next are absent. In contrast, the preferred directions of single motion detectors are likely to correspond to the distinct axes of the ommatidial lattice [30]. The smoothness of shifts in direction tuning across dendritic branches therefore suggests that the measured local dendritic activity represents pooled signals from several motion detectors instead of single inputs. Alternatively, the smoothness of shifts might result from the fact that the ommatidial lattice orientations themselves shift across the eye, resulting in spatial differences in the alignment of the stimulus with the ommatidial lattice [30].

## Conclusions

By using calcium imaging to visualize dendritic activation during visual motion in various directions, we were able to obtain a direct view at the local variations in directional selectivity of the dendrites of individual fly LPTCs. These dendritic response patterns form a filter for the evaluation of complex motion patterns in large parts of the visual field of the animal as may be induced during different types of flight manoeuvres. Our experiments show that in the fly LPTCs differences in local preferred directions are maintained on the level of cytosolic calcium concentration changes across the dendrites. Thus, these neurons are capable of providing their postsynaptic targets with a matrix of spatially varying outputs via dendritic synapses or, alternatively, with integrated output via single axonal output synapses. However, rather than simply integrating inputs in a purely retinotopic way, some LPTCs also exhibit local variations in direction selectivity that are the result of indirect non-retinotopic inputs, which converge via extended synapses from the contralateral brain hemisphere. Even though individual motion detector inputs still remain to be uncovered, this direct insight into processes which take place on the dendrites of these cells is a first step to a more detailed understanding about how motion signals are integrated and processed by single neurons in order to generate behaviourally relevant output information.

## Methods

### Flies

Blowflies (*Calliphora vicina*) were raised in the department's stock at 25°C in a 12 h light/12 h dark cycle. Experiments were carried out on females collected <3 days after eclosion.

### Preparation

After affixing the fly's thorax in a horizontal position to a glass cover slide, legs, antennae, proboscis and the digestive tract were removed and the openings in the cuticle closed with beeswax. The head was pulled downwards and attached to the thorax to allow access to the head capsule. An opening was cut into the right half of the caudal head cuticle and a hole was cut into the dorsal thorax to insert the reference electrode. The exposed tissue was supplied with insect ringer solution [29] to prevent desiccation.

### Intracellular recording

Sharp borosilicate glass electrodes (G100TF-4, Warner Instruments, CN, USA) were pulled on a Brown-Flaming P-2000 Puller (Sutter Instruments, CA, USA) in order to create resistances of 95 - 110 MΩ when filled with Oregon Green/1 M KCl. A glass reference electrode was placed in the fly's thorax and connected to a supply of insect Ringer's solution, which also served as the medium for water immersion microscopy when needed. Recording was done in the right lobula plate. Electrode signals were amplified by a factor of 10 using a custom-built amplifier. Responses were sampled at 4000 Hz (DT2801A, Data Translation, MA, USA) and stored on hard disk for offline analysis.

### Calcium Imaging

For the visualization of calcium signals, we filled the tips of the recording electrodes with 15 mM Oregon Green 488 (BAPTA)-1 hexapotassium salt (Molecular Probes, OR, USA) in 1.7 mM KOH/33 mM (4-(2-hydroxyethyl)-1-peperazineethanesulphonic acid/3.3 mM KCl. The dye was injected by passing hyperpolarising currents of 0.6 to 1 nA for 7-20 min (varying between experiments) during the determination of cell type by means of electrophysiological recordings. Relative cytosolic Ca^2+ ^concentration changes were monitored by epifluorescence imaging of Oregon Green emission using a dry Leica HC PL Fluotar 10× and a water immersion Olympus LUMPlan FI/IR 40× objective at an upright fixed-stage microscope (Leica DMLFSA, filter set: excitation BP 470/40 nm, dicroic mirror 510 nm, emission LP 515 nm and BP 530/50 nm) equipped with an electron-multiplying charged-coupled device camera (Andor iXon DV887-BI, Andor Technology PLC, Belfast, Northern Ireland), operated at frame rates of 10 to 26 Hz and at a resolution of 512 × 512 pixels (pxs). A 488 nM laser (FiberTEC, Blue Sky Research, CA, USA), diffused by a frosted glass, was used as an excitation light source for one-photon wide-field imaging. For multifocal two-photon imaging, we used a tuneable Mai-Tai infra-red laser (Spectra-Physics, CA, USA) at 800 nm and a multifocal beam splitter (TriM-Scope, LaVision BioTec, Bielefeld, Germany) set to eight excitation foci covering a scan area of 357 × 254 px/102 × 73 μm (for details see [29]). During the recordings, no noticeable bleaching could be found. Usually the reason for terminating a measurement series was a fairly abrupt deterioration of the visually induced calcium response.

### Visual stimulation

A board of 22 × 45 green light-emitting diodes (LEDS; each measuring ~4.8 × 2.5 mm, emission maximum at ~570 nm, covered with a LP550 filter in order to reduce interference with fluorescence emission light) was used to simulate a moving high contrast square wave pattern (temporal frequency: 4 Hz, spatial frequency: 10°, mean luminance: ~30 cdm^-2^). The visible pattern consisted of an octagonal area centred in the frontal visual field with an angular extent of ~80 × 80°. The LED plate could be pivoted around the centre, allowing us to change the direction of motion in 45°-steps whilst leaving the visible area constant. Stimulus directions were changed pseudorandomly (sequence: 180°-0°-135°-315°-90°-270°-45°-315° or reverse). In some experiments half of the pattern was covered, leaving only the ipsilateral or contralateral part visible. In these series, which were recorded in consecutive blocks for technical reasons, the same stimulus sequence was used in ipsi- and contralateral stimulation to enhance comparability.

### Data analysis

Camera control and image acquisition were performed using ImSpector 3.20 (LaVision Biotec). Routines written in C (Borland, CA, USA) were used to control visual stimulation and electrophysiological data acquisition. Frame triggers from the camera were used to synchronize laser scanning, image acquisition, visual stimulation and voltage recording. Matlab (The Mathworks, MA, USA) was used for data analysis. Ca^2+ ^concentration signals were evaluated as background-subtracted pixel-wise changes from baseline levels of the fluorescence of the Ca^2+^-sensitive dye divided by the baseline value (Δ*F*/*F*_0_). To compensate for global fluctuation in image brightness, mean intensity values of a reference area outside the dendrite were taken and subtracted from the signal. Baseline values F_0 _were calculated from the mean of the first three images in the series. For selectivity arrow plots, single direction response amplitudes were calculated by averaging Δ*F *during stimulus duration. For the calculation of the resulting vector, length and direction were calculated by vector addition of the eight vectors representing single directions, while brightness was calculated by scalar addition of the absolute values for the eight single direction responses.

## List of abbreviations

Amx: amacrine cell; BAPTA: 1,2-bis(o-aminophenoxy)ethane-N,N,N',N'-tetraacetic acid; EMCCD: electron-multiplying charge-coupled device; HS: horizontal system; LED: light emitting diode; LPTC: lobula plate tangential cell; px: pixel; ROI: region of interest; TPLSM: two-photon laser-scanning microscopy; vCH: ventral centrifugal horizontal; VS: vertical system.

## Authors' contributions

CS performed the experiments, analysed the data and drafted the manuscript. RK conceived the concept of the study and contributed to interpretation of data and writing the manuscript. PT participated in the technical design of the experiments. ME was involved in interpretation of data and writing the manuscript. All authors have read and approved the final manuscript.
